# Adopting the YOLOv4 Architecture for Low-Latency Multispectral Pedestrian Detection in Autonomous Driving

**DOI:** 10.3390/s22031082

**Published:** 2022-01-30

**Authors:** Kamil Roszyk, Michał R. Nowicki, Piotr Skrzypczyński

**Affiliations:** Institute of Robotics and Machine Intelligence, Poznan University of Technology, 60-965 Poznan, Poland; kamil.roszyk22@gmail.com (K.R.); piotr.skrzypczynski@put.poznan.pl (P.S.)

**Keywords:** pedestrian detection, multispectral fusion, deep learning, You Only Look Once, real-time

## Abstract

Detecting pedestrians in autonomous driving is a safety-critical task, and the decision to avoid a a person has to be made with minimal latency. Multispectral approaches that combine RGB and thermal images are researched extensively, as they make it possible to gain robustness under varying illumination and weather conditions. State-of-the-art solutions employing deep neural networks offer high accuracy of pedestrian detection. However, the literature is short of works that evaluate multispectral pedestrian detection with respect to its feasibility in obstacle avoidance scenarios, taking into account the motion of the vehicle. Therefore, we investigated the real-time neural network detector architecture You Only Look Once, the latest version (YOLOv4), and demonstrate that this detector can be adapted to multispectral pedestrian detection. It can achieve accuracy on par with the state-of-the-art while being highly computationally efficient, thereby supporting low-latency decision making. The results achieved on the KAIST dataset were evaluated from the perspective of automotive applications, where low latency and a low number of false negatives are critical parameters. The middle fusion approach to YOLOv4 in its Tiny variant achieved the best accuracy to computational efficiency trade-off among the evaluated architectures.

## 1. Introduction

Reliable real-time detection and recognition of other road users enable safe maneuver planning and execution in autonomous driving. Among those other traffic participants, pedestrians are considered the most vulnerable road users. According to the World Health Organisation (WHO) reports [1], about half of the death casualties of road accidents per day are vulnerable road users. Therefore, there is a need to develop more advanced pedestrian detection systems for autonomous driving. The most common sensors in autonomous vehicles are passive RGB cameras [2] that are vulnerable to changes in lighting conditions. Hence, additional information from thermal cameras operating in the infrared spectrum [3] is often used to detect pedestrians. As thermal images also have shortcomings related to the lower resolution than RGB cameras and a lack of background textures, multispectral fusion methods have attracted attention in recent years.

Current technologies for considering pedestrians in the design of an autonomous vehicle were recently surveyed [4]. While in this survey, the technologies for handling pedestrians were found to be mature, there is still a gap between the recent advances in general-purpose object detection and recognition [5], and the solutions for multispectral pedestrian detection proposed so far. Namely, although the state-of-the-art solutions to this problem use deep neural networks and propose elaborated neural architectures that exploit the complementary visual information, the high latency (for inference generation) of the neural models often prevents these systems from producing an useful responses in time, thereby not allowing the whole control system to make a good decision. Moreover, the applications for autonomous cars are limited by the GPUs available in the vehicles themselves, and the available processing power is used to perform multiple perception tasks. Therefore, in this article, we investigate the adoption of the real-time neural network detector You Only Look Once (YOLO) [6], specifically its most recent version YOLOv4 [7], for the task of multispectral pedestrian detection. Our aim is to design a neural network architecture that exhibits detection accuracy on par with the state-of-the-art but is more efficient with respect to the inference time, thereby allowing the control system of an autonomous vehicle to make a decision while still far enough away from a vulnerable road user.

We re-visit the general fusion paradigms that were already investigated for pedestrian detection with the Faster R-CNN architecture [8]: early fusion at the level of images, late fusion at the level of detection (bounding boxes), and middle fusion at the level of features. Then, we recommend the fusion scheme that provides the best trade-off between the accuracy in various scenarios (day and night) and latency. The obtained results are further refined in the lightweight configuration of a YOLOv4-Tiny model optimized with Nvidia’s TensorRT tool. We report the results of experiments on the KAIST multispectral pedestrian detection dataset [9], which is commonly used to benchmark pedestrian detection methods, but propose a new evaluation measure capturing the viability of the method for deployment in the control system of an autonomous vehicle. The contributions of this article can be summarized as:

- We investigated how the detection frame rate (expressed in frames per second, fps) influences the recall measure in a realistic scenario wherein the goal is to detect a person and brake before a collision occurs. This analysis allowed us to conclude that low latency during detection is a key factor in pedestrian detection. We ought to increase speed of detection algorithms so they can spot pedestrians and initiate safe breaking in time.
- In the context of a realistic scenario, we investigated five different fusion schemes for multispectral images that were inspired by the state-of-the-art, but are our original contributions to the YOLOv4 architecture. These fusion schemes range from very simple early fusion at the level of image data to elaborated middle and late fusion schemes.
- As a result of those investigations, we developed a new YOLOv4-based architecture that allows for middle fusion and scored the best on average in the experiments while processing the multispectral images at 35 fps.
- Being aware of the limited computing resources of autonomous cars, we prepared a lightweight model. This detector exceeds 400 fps on the desktop Nvidia RTX 3080 GPU, provides the lowest latency when detecting vulnerable road users from a moving vehicle, and can be deployed on edge computing devices.

The remainder of this article is organized as follows. Related works are reviewed in Section 2. Section 3 introduces the YOLOv4 architecture and demonstrates how it accomplishes the task of pedestrian detection on both RGB and thermal images. Section 4 describes the proposed multispectral fusion approaches using the modified YOLOv4 networks. Next, Section 5 provides the experimental results and performance assessment. Section 6 contributes an in-depth analysis of the detector’s performance in the context of an application in autonomous driving. Finally, Section 7 discusses the conclusions and future work.

## 2. Related Work

### 2.1. System Architectures for Pedestrian Detection

Traditional object detection techniques use hand-crafted features and their descriptors to determine the properties of objects in an image. Such techniques applied to pedestrian detection on visible light images [10] have included Histogram of Oriented Gradients (HOG) [10] and Local Binary Patterns (LBP) [11] features, with the Integral Channel Feature (ICF) detector [12] being the most successful [13]. As hand-crafted features limit the performance of a detector [13], convolutional neural networks (CNNs) can be applied to extract features automatically.

Object detection algorithms, which use CNNs, fall into two main types: two-stage and single-stage detectors. Typical two-stage detectors come from the region-based convolutional neural network (R-CNN) family. In the Faster R-CNN [14] version, the region proposal network (RPN) was introduced, which can predict the bounding box and score at each position simultaneously, resulting in a substantial reduction in the prediction time. Deep learning methods based on variants of the R-CNN architecture are widely used to detect pedestrians in visible light images [15,16], but the Faster R-CNN architecture was also successfully adapted for the fusion of visual and thermal images in pedestrian detection [8,17,18]. Although the architectures of two-stage detectors were substantially improved concerning their computational efficiency, these neural networks cannot process images at the full resolution of a camera and the required frame rate. Therefore, only single-stage detectors can be considered for tasks that require real-time operation.

The most widely used single-stage CNN architectures belong to the You Only Look Once (YOLO) family. The YOLO architecture [6] treats object detection as a regression problem. Although the regression-based detectors are considered less accurate than their region-proposal-based counterparts, they are significantly faster [19]. The YOLO concept was further improved by its authors [20,21], and the latest version created by Bochkovskiy et al. [7] comes with changes that speed up the algorithm even more and improve detection accuracy. The YOLO architecture outperforms two-stage detectors concerning the computational speed, which typically results in a higher frame rate in image processing while detecting objects. However, the memory requirements of the Darknet backbone network in YOLO are too demanding for embedded devices, and the resulting processing speed is insufficient in some applications. Hence, several scaled-down YOLO variants, often referred to as “YOLO-Tiny,” have been proposed [22]. Variants of YOLO-Tiny have already been applied in the context of automotive applications, demonstrating high performance in object detection [23,24]. The YOLO family networks were already employed in pedestrian detection with visible light images [25], and recently, YOLO-based network architectures were proposed for pedestrian detection using multispectral fusion. The MAF-YOLO approach [26] adapted YOLOv3 architecture. The approach in [27] adopts the most recent YOLOv4 version, which was also leveraged in our research. Furthermore, other single-stage neural network detectors were investigated for multispectral pedestrian detection: the Single Shot Detector (SSD) [28] and the Central and Scale Prediction Network (CSPNet) [29].

### 2.2. Multispectral Fusion in Pedestrian Detection

The methodology of multi-sensory fusion using deep learning methods in the context of automotive applications was presented in a recent survey [30]. Implementing an effective fusion scheme requires addressing three points: what to fuse, when to fuse them, and how to fuse them.

The first question concerns the sources of information that are most relevant to the task at hand. In the pedestrian detection task, combining regular RGB images and thermal images acquired in the infrared spectrum improves the reliability of detection. The RGB and thermal sensing modalities perform best under different scenarios, with the RGB images yielding precise cues related to visual details, if the lighting conditions permit, whereas thermal images recover cues related to pedestrians in deep shadows and night-time scenes [3,31,32].

The second question relates to the processing stage when the information is being fused into a coherent structure that makes it possible to decide if a pedestrian is detected or not. In general, the information about pedestrians conveyed in the visible and thermal modalities can be combined at the level of images, or the level of detection, in the form of locations of the bounding boxes produced from two distinct processing pipelines [33]. This led to the early and late fusion paradigms, which were compared in the early works on deep-learning multispectral fusion for pedestrian detection [18]. The popular Faster R-CNN detector was adapted to multispectral pedestrian detection in [8,18,33], investigating fusion at different stages of the neural processing architecture. The work of Liu et al. [8] concludes that the fusion of visible and thermal features at the middle stage of processing outperforms both more obvious early and late fusion schemes.

The last question concerns the ways of performing sensory fusions and is closely related to the transformation of input information inside the network. The standard feature fusion methods include the addition or concatenation of feature maps, but more complicated procedures are also applied. Such methods can exploit additional context information, e.g., produced by semantic segmentation [34], or weight the detection results from the visible and thermal parts according to the prediction of the illumination conditions [33,35]. The last few years brought interesting results [31,32] demonstrating that adaptive, learning-based fusion schemes outperform hand-crafted fusion mechanisms in the multispectral pedestrian detection task. These schemes work at the middle level but introduce some attention mechanisms [36] to the network to assign weighting factors to feature maps obtained from the different input modalities. The attention module in [32] works at the channel level, considering the bounding box information and distinguishing between the background and pedestrians in an image. The more recent approach presented in [31] adds a guidance mechanism for inter-modality attention, which selects RGB or thermal features according to a mask, providing a dynamic comparison of their importance.

Recently, multispectral fusion methods for pedestrian detection have been implemented using single-stage neural network detector architectures, such as the Single Shot Detector (SSD) [28] and the Central and Scale Prediction Network (CSPNet) [29]. Furthermore, the YOLO architecture has been adapted for pedestrian detection fusing RGB and thermal images. The MAF-YOLO approach [26] adapted the YOLOv3 architecture, improving the Darknet53 backbone network to work better with infrared images of small objects, and adding an attention mechanism for better accuracy. The works of Cao et al. [27] and Dao et al. [37] leverage the most recent YOLOv4 version for multispectral pedestrian detection. The research presented in [27] investigated several fusion schemes at different stages of the neural processing pipeline, and selected the middle fusion scheme as the most effective, which corroborates our findings, and makes that work most similar to ours. The best solution from [27] had similar performance to our YOLO4-Middle, with a slightly better detection accuracy but slightly longer inference time. However, in [27] the performance was measured only in terms of the log average miss rate for the two modalities, and they did not attempt to investigate how the performance impacts the ability to detect pedestrians quickly in day and night scenarios. We demonstrate in Section 6 that low latency of the detector (i.e., very short inference time) is a key factor in ensuring safe breaking when pedestrians are detected. Another recent study that leveraged YOLOv4 [37] involved a different approach to fusion, with an architecture that deploys two YOLOv4 pipelines for separate processing of RGB and thermal images, and then fuses the results using weights computed upon the estimated illumination condition. The architecture proposed in [37] requires the processing of both images through the entire YOLOv4 pipeline, which results in a processing speed that is clearly inferior to that of our solution (16 fps vs. 35 fps, respectively), though their accuracies are comparable. With respect to state-of-the-art, we were also the first to investigate a scaled-down "tiny" variant of the YOLO architecture for the detection of pedestrians. We found that although the accuracy of detection (in terms of mean average precision) drops for the scaled-down architecture, the extremely short inference time allows our YOLO4-Tiny-Middle to score best when the ability to provide reliable detection of distant pedestrians in a car breaking scenario is assessed.

## 3. Pedestrian Detection with YOLOv4

Pedestrian detection for intelligent vehicles requires low latency of information to make steering decisions on time while also performing inferences several times per second to observe the changing environment. Therefore, we selected YOLOv4 as a baseline, state-of-the-art network that meets these requirements. We start the introduction of our approaches with a classical RGB image-based YOLOv4 detector, as described in Section 3.1, called YOLO4-RGB. This baseline approach was adapted to work with thermal images only as explained in Section 3.2 and called YOLO4-T.

### 3.1. YOLOv4 with RGB Images

**Figure 1:**
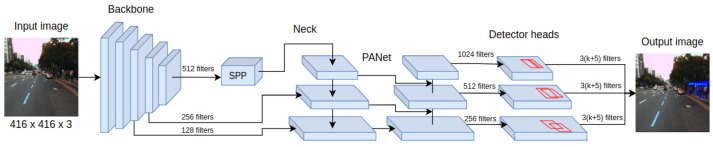
Scheme showing the main processing components of the YOLOv4 detector architecture.

The main requirements for developing pedestrian detection systems for autonomous vehicles are high recognition accuracy and the ability to operate the system in real-time. Those requirements are fulfilled by the YOLOv4 detector, which belongs to the family of single-stage detectors. Among the key features that distinguish YOLOv4 from earlier versions is the detector architecture, consisting of a backbone, neck, and head as presented in Figure 1. As the backbone, the CSPDarknet53 is used. This convolutional neural network contains residual connections preventing gradient vanishing and allowing information to flow from the initial to the final layers.

The detector layers that are directly connected to the backbone are called the neck, and for YOLOv4, they take the form of PANet (Path Aggregation Network). Their task is to extract feature maps at various stages of backbone processing, allowing more efficient detection of objects of different sizes. What is more, SPP (Spatial Pyramid Pooling) is also often included as part of the neck, which extends the receptive field of the detector, increasing its final accuracy. This is possible thanks to performing max-pooling operations with different kernel values for given feature maps. The final components of the YOLOv-4 detector are its heads, which have not changed since the previous version. Their multiplicity allows them to be combined with layers of various feature extraction levels, which helps detect objects of different sizes. Important also is the collection of techniques called Bag of Specials—low computational cost modules for both the backbone and the detector of the YOLOv4 architecture, including the new activation function Mish.

### 3.2. YOLOv4 with Thermal Images

RGB cameras are commonly used in scenarios assuming observations in the visible light spectrum. However, these cameras perform poorly in poor lighting conditions, such as during adverse weather conditions or at night. Moreover, poor performance can also be observed during rapid changes in illumination or when a single source of light, e.g., the sun, blinds the camera.

Capturing the light spectrum at different wavelengths provides different views of the scene. If we decide to capture information at wavelengths up to 14 μm, we get a thermal camera. Thermal cameras perform their measurements in the infrared spectrum, observing objects’ heat emissions. Thermal images are relatively independent of lighting conditions, making thermal cameras effective at detecting objects that are invisible to RGB cameras.

In our experiments, we used the 1-channel, 8-bit thermal data encoding initially provided by the authors of the KAIST dataset, which we normalized with a histogram to use the whole range of 8-bit channel information. The 8-bit thermal data encoding instead of raw 14-bit representation is a known limitation of this dataset [38]. As a result of data preprocessing, we obtained thermal data compatible with the original YOLO4-RGB architecture, not requiring modifications to the architecture itself. The version that works with thermal data was dubbed YOLO4-T, with the T standing for the thermal input. The internal layers of the network were not changed, and the network was pretrained on the COCO dataset, similarly to YOLO4-RGB.

## 4. Sensory Fusion with the YOLO Architecture

Performing the fusion of different sensor modalities requires proper alignment of these measurements, understood as finding the correspondence between individual measurements from each sensor. We assumed that the RGB and thermal images are aligned, based either directly on the hardware used to capture the sensory data [9] or by additional processing steps [39]. Once calibrated, we could consider different approaches to fusing the information in the deep learning-based processing pipeline. We decided to differentiate between these approaches based on the moment when information from different sensing modalities is combined in the detection processing pipeline. Our approaches were divided into three main groups: early fusion, late fusion, and middle-fusion methods.

### 4.1. Early Fusion Approaches

Early fusion approaches focus on combining the RGB and thermal images on the raw data level prior to being processed by the neural network. In our case, it means that the RGB and thermal data were packed into a single, multi-channel image that was passed to the network for further processing. In these approaches, the latent features created in the backbone of YOLO contain information from both cameras from the first computational stage. The main idea is that the input streams are complementary at the low level of processing. The deep learning network can determine which input properties are essential for object detection.

The original network architecture of YOLO processes the three-channel image as an input. Therefore, we divide our approaches into subgroups of methods that preprocess the initial data to fit these input types (YOLO4-HST, YOLO4-GST) and a method that modifies the network architecture to take a four-channel image as an input (YOLO4-RGB-T).

#### 4.1.1. Yolo4-HST and YOLO4-GST Fusions

Providing the RGB and thermal images as a three-channel input means some information must be compressed to fit the input requirements. In the two proposed methods, we convert the image in RGB color space into a different color representation and select two channels containing the essential information for pedestrian detection.

In the YOLO4-HST approach, the RGB color space is converted into the HSV (hue, saturation, value), also known as HSB (hue, saturation, brightness). Then, the HST space is created using the hue, saturation, and thermal components. In this representation, the brightness component is omitted. The YOLO4-HST is trained and evaluated on the input data converted into HST space.

**Figure 2:**
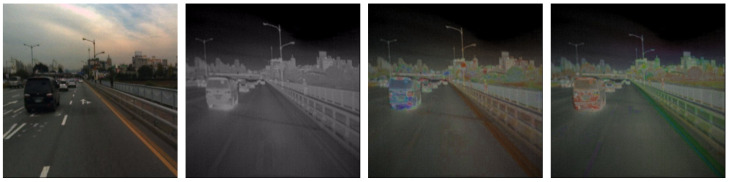
Example input images processed by the YOLOv4 architecture: RGB and thermal images on the left and HST and GST images on the right.

In the YOLO4-GST approach, the GST representation consists of the G channel, which is the color image transformed to grayscale, containing the input from all RGB channels; the S channel, which is the saturation from the HSV representation of the color image; and the T channel which is the grayscale thermal image. Exemplary images converted into HST and GST representations are presented in Figure 2.

#### 4.1.2. YOLO4-RGB-T Fusion

**Figure 3:**
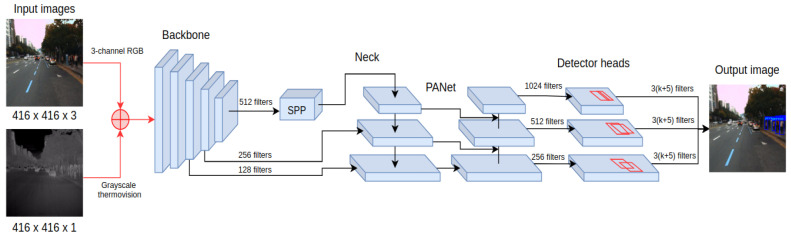
The processing pipeline of the YOLO4-RGB-T network combines RGB and thermal images into a 4-channel input.

YOLO4-RGB-T fusion is an early fusion approach that modifies the input of the deep learning network. In this approach, the 4-channel image is taken as an input and consists of a 3-channel RGB image and 1-channel thermal information, as presented in Figure 3. As RGB and thermal information is fed directly to the network, no information is lost during data conversion.

The modified architecture of YOLOv4 has an extra input dimension for the first convolutional layer, which raises the total number of parameters of the network when compared to YOLO4-RGB or YOLO4-T. Moreover, direct weight transfer from the original YOLO4-RGB is not feasible. Nevertheless, we decided to use pretrained weights from YOLO4-RGB for all common parts between architectures. To increase the robustness of the learning procedure, we first trained the weights of the modified input layer for the first two epochs with the weights of the remaining layers (frozen). The duration of two epochs was chosen experimentally based on the learning curve to provide a good bootstrap of the network’s weights while avoiding convergence to a local minimum. Once the first two epochs were completed, the weights in the whole network were trained for the remaining 23 epochs. In total, the training process lasted for 25 epochs.

### 4.2. Late Fusion Approach (YOLO4-Late)

**Figure 4:**
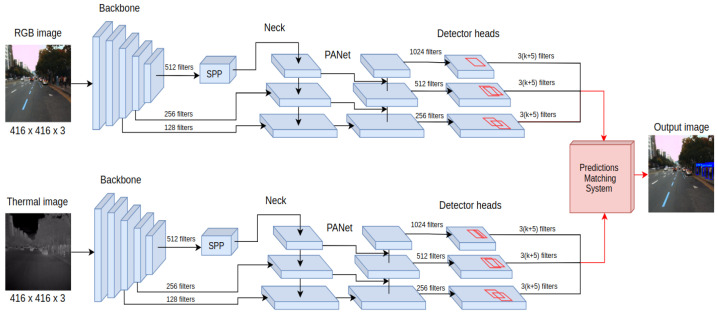
The scheme shows the YOLO4-Late detector architecture, using a prediction matching system to provide unified pedestrian detections based on the inputs of both network branches.

The previous section proposed a neural network that performs the fusion scheme at the data input level. An entirely different approach is to perform independent processing of the information coming from RGB and thermal cameras while fusing the processing results. In this fusion approach, called YOLO4-Late and presented in Figure 4, the deep neural network consists of YOLO4-RGB to process an RGB image and YOLO4-T to process the thermal image.

The detection results obtained from both networks are processed by a proposed, non-learnable component, which we call the prediction matching system (PMS). The responsibility of the PMS is to provide a single detection output based on the results obtained from both processing branches, thereby fusing the results of both networks. In YOLO4-Late, the PMS takes as an inputs the value of the intersection over union (IoU) parameter for bounding boxes for the outputs of both neural networks and the probability that a detected object belongs to a given class calculated by each network. In the proposed implementation, the PMS follows a preset rule that reports detection of an object if the IoU value for the predictions from both detectors is greater than 0.6 or if the probability of detection for either of the networks is greater than 0.45. The provided thresholds for the IoU value and probability detection were determined with a simple exhaustive search on the training data to provide the best mAP measure.

### 4.3. Middle Fusion Approach (YOLO4-Middle)

**Figure 5:**
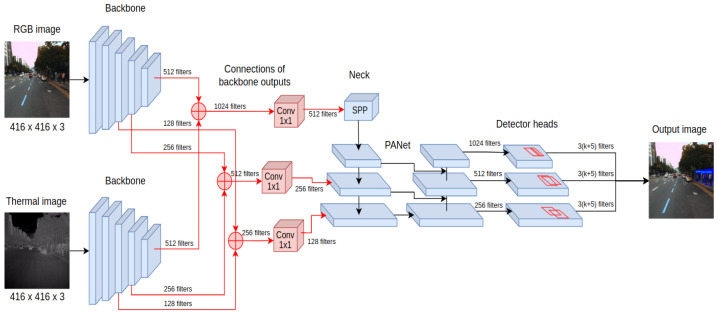
The processing pipeline of the YOLO4-Middle detector architecture extracts independent features for RGB and thermal inputs that are combined on different processing levels before the layers responsible for object detection.

The last proposed method, YOLO4-Middle, performs the fusion on the feature-level processing of the input. In this configuration, the input is a 6-channel tensor input, where three channels are filled with RGB image data, and three channels contain the thermal image. In the beginning, the processing is performed independently for RGB and thermal inputs using the CSPDarknet53 neural network—see the initial feature processing presented in Figure 5. This ensures that a separate pipeline preprocesses each image with a different domain to determine the feature maps. In contrast to previous fusion approaches, these feature maps are combined at different processing levels using inter-branch connections.

The feature maps obtained from processing RGB and thermal inputs are concatenated and then processed using convolutional layers with a kernel size of 1 × 1. This operation reduces the depth of the feature maps, making it possible to keep the remaining processing layers of the YOLOv4 detector architecture intact compared to processing with a single sensory input.

The proposed architecture of YOLO4-Middle was initialized with original pretrained weights for the YOLO4-RGB detector for all the processing blocks that are the same as those of the original pipeline. However, similarly to YOLO4-RGB-T, some components differ, and the weights had to be trained without initial guesses. Therefore, the neural network training was split into two stages—a warm-up and the main training. The warm-up lasted for two epochs and was used to determine the initial values of weights that could not be initialized with transfer learning, i.e., for new convolutional layers that combined the output feature maps from both sources. The transferred weights were frozen during the warm-up stage of training. The main training started from the third epoch when all the weights in the network were trained.

## 5. Experiments

### 5.1. Kaist Dataset

An experimental evaluation of the proposed networks that fuse the RGB and thermal inputs in pedestrian detection requires a dataset that recorded from a moving car that contains aligned images from both sensor modalities. The KAIST dataset [9] meets these requirements, as RGB and thermal cameras were placed on a unique sensory setup on the car’s rooftop. This sensory setup allows recording images from both sensors while ensuring that each pixel of the RGB image corresponds to the same pixel of the thermal image. In practice, it means that data from both sensors are aligned, and the proposed sensory fusion approaches can be directly used.

The dataset consists of over 60,000 images recorded at different times of the day. The authors of the dataset divided these sequences into day and night parts. Each recording has object annotations defined by the authors of the dataset, which can be used during training and to evaluate the proposed system. The ground truth labels are available for three classes: a person, a group of people, and a cyclist. In our approach, we merged all of these classes into a single class “person”, as the dataset was not balanced, and each annotated class represents a person in the end.

Each sequence in the KAIST dataset was used only during the training, validation, or testing phase, which avoided the overlap between data. This approach ensured that the detector would not know the test data, allowing for an unbiased prediction of its accuracy. Table 1 shows the division of the dataset into training, validation, and test sets, together with the number of objects included in the image sequences.

### 5.2. Precision Performance Comparison

**Figure 6:**
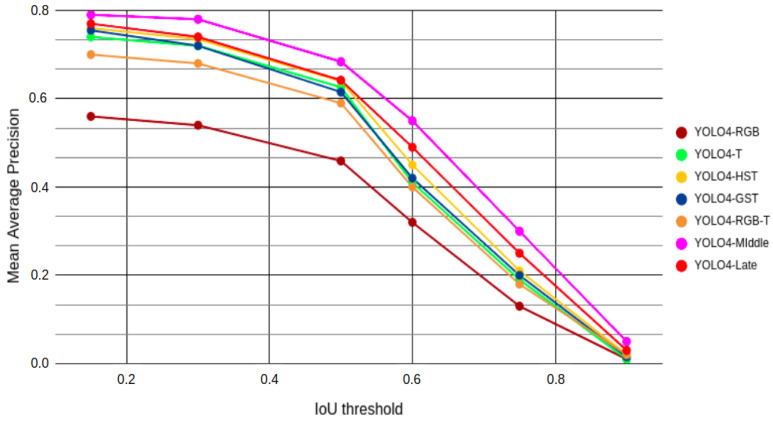
Measured mAP for different IoU threshold values for all considered fusion approaches.

The typical way of measuring the detection performance is based on the mean average precision (mAP). The mAP metric determines how many of the predictions turned out to be correct and what was the overlap between the detected and ground truth locations of the objects. The mAP is therefore useful for comparing the performances of different detection algorithms. Furthermore, the values of mAP are strongly connected with the preset threshold that determines the overlapping of the predicted bounding boxes with the ground truth ones. The overlap threshold is denoted by the Intersection over Union (IoU) value. The lower the IoU is, the lower overlap is needed to be considered correct, increasing the number of detections that are assumed to be accurate. In our case, we computed mAP values for IoU thresholds of 0.15 $\left(mAP_{15}\right)$, 0.30 $\left(mAP_{30}\right)$, 0.50 $\left(mAP_{50}\right)$, 0.60 $\left(mAP_{60}\right)$, 0.75 $\left(mAP_{75}\right)$, and 0.90 $\left(mAP_{90}\right)$ for all considered fusion approaches. The obtained performances for all considered methods are presented in Figure 6.

From this visual representation of the mAP values for all IoU thresholds, the highest metric scores were reached by the YOLO4-Middle detector, which combines extracted information from two sources at different processing levels. Slightly worse results were achieved by the YOLO4-Late detector, which combines the detection results from parallel processing pipelines for RGB and thermal images.

Marginally worse results can be observed for the YOLO4-GST and YOLO4-HST fusions, which barely outperformed the detector processing only the thermal images (YOLO4-T). Despite using all available information, similar but slightly lower performance than for YOLO4-T can be observed for YOLO4-RGB-T. We believe it might have been caused by a partial inability to use weight transfer learning to boost the initial training of the network for YOLO4-RGB-T. The worst mAP metric values were obtained by the YOLO4-RGB detector, proving the importance of performing data fusion for data from RGB and thermal domains.

**Figure 7:**
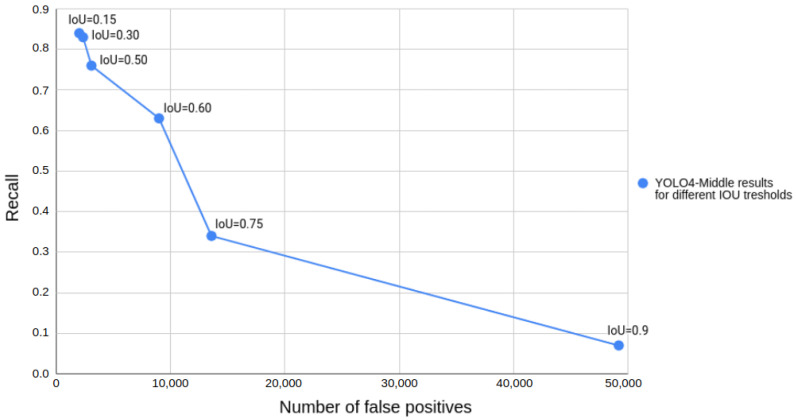
The obtained plot of recall and number of false positives depending on the chosen IoU threshold for YOLO4-Middle.

The ranking of the considered approaches is almost the same regardless of the chosen IoU threshold. However, a single IoU threshold value has to be chosen during the operation. To select this value, we decided to analyze the plot of recall and false positives depending on the IoU threshold for YOLO4-Middle, as presented in Figure 7. We did not use the popular receiver operating characteristic (ROC) curve, as for the object detection task it is problematic to count the proper number of true negatives. In our case, we are interested in detecting the pedestrian as soon as possible to determine if emergency braking can be performed. Therefore, we are less concerned about the accuracy of the bounding box detection and more interested in a greater recall value. We conclude that a lower value of the IoU threshold would be preferred in this scenario, as long as we would be able to filter the false positives. We finally chose the IoU of 0.50 as a good trade-off and the value typically used in state-of-the-art works on pedestrian detection. Therefore, for further analysis we focused on the IoU of 0.50. The $mAP_{50}$ results obtained by the methods, depending on the time of the day, are presented in Table 2.

As previously seen in Figure 6, the best overall performance according to $mAP_{50}$ metric was obtained by YOLO4-Middle, which significantly outperformed the other methods on the day images. This difference in performance is especially evident when compared to the single-source approaches, such as YOLO4-RGB and YOLO4-T: YOLO4-Middle had more than 0.066 $mAP_{50}$ improvements over both of those methods. For night conditions, YOLO4-Middle outperformed YOLO4-RGB by 0.328 and YOLO4-T by 0.009. This proves that the middle data fusion can improve performance in all conditions compared to single modality approaches. It is worth noting that the YOLO4-Late detector obtained the best performance at night, achieving a $mAP_{50}$ measure 0.010 higher than YOLO4-Middle.

The remaining fusion approaches had similar performances for day and night conditions, showing that fusion methods can perform well regardless of these conditions, even though YOLO4-RGB performs very poorly in night conditions.

### 5.3. Performance as a Function of Object Size

**Figure 8:**
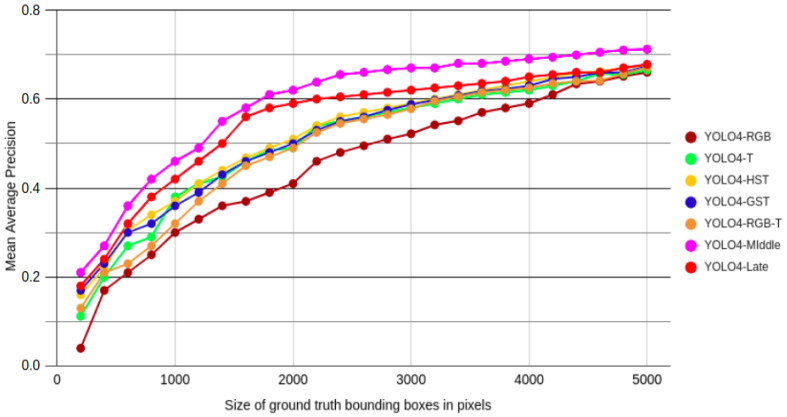
The plot of the $mAP_{50}$ metric for different sizes of ground truth bounding boxes in a range from 200 to 5000 pixels and different configurations of the proposed detector.

The results show that the YOLO4-Middle architecture was the best performing network among the verified solutions according to the mAP measure. A single mAP value is insufficient to determine the method’s viability to be deployed in real-world scenarios. Therefore, we decided to additionally analyze the performances of the presented approaches based on the ground truth pixel sizes of the objects. The ground truth pixel size of an object is defined as the sum of pixels that were used to mark the object in the dataset on the particular images. Then, we computed the mAP performance of the network for all images in the testing part of the dataset for a considered bounding box size. The $mAP_{50}$ results obtained for all considered methods and different sizes of objects are presented in Figure 8.

In Figure 8, it can be seen that the performances of all of the methods drop when objects get smaller. For all considered scenarios, the best performance can be seen for YOLO4-Middle, with $mAP_{50}$ reaching nearly 0.700 for objects larger than 4000 pixels; but $mAP_{50}$ dropped to below 0.300 for the smallest objects. Right behind it, the YOLO4-Late detector also achieved the highest mAP values for the largest objects. The results confirm that fusion techniques are essential for detecting distant objects, often at a significant distance from the vehicle.

### 5.4. Inference Time

In automotive applications, we are interested in obtaining the best possible results considering real-world scenarios. While the vehicle is in motion, the system has a limited time budget to perform efficient detection and decide, i.e., to perform emergency braking. The obtained processing fps values for the analyzed fusion models are presented in Table 3, and the inference times were computed assuming that each image was processed independently. The optimized models were obtained after processing original networks through Nvidia’s SDK for high-performance deep learning inference, TensorRT (https://github.com/NVIDIA/TensorRT accessed on 20 November 2021).

After optimizing neural network models, the processing times for all analyzed methods dropped below 30 ms for a single image. The longest inference time was observed for YOLO4-Middle, which has additional connections between processing blocks for RGB and thermal pipelines, leading to a long processing time. As it turns out, the middle fusion architecture provides the best results concerning the accuracy of pedestrian detection in both day and night scenarios. Still, it is slower than the completely independent processing pipelines for both modalities for the YOLO4-Late approach. Although the achieved speed of 35 fps satisfies our assumptions of low latency, as we can process the incoming images essentially at the fps of a typical RGB camera, this was achieved using the total processing power of the Nvidia RTX 3080 GPU.

### 5.5. The Lightweight Approach: YOLO4-Tiny-Middle

**Figure 9:**
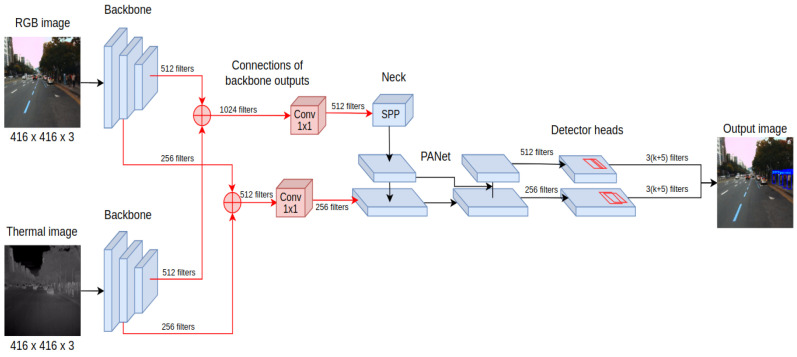
The processing pipeline of the YOLO4-Middle-Tiny detector. It extracts independent features from RGB and thermal inputs that are combined on different processing levels before the layers responsible for object detection.

Considering the speed versus accuracy trade-off, we decided to extend our investigations to the tiny-YOLO architecture, which is known for its superb computational efficiency. We considered our YOLO4-Middle fusion scheme as the baseline for further development, as it performed well for the day, night, and smaller objects. We created the YOLO4-Tiny-Middle version starting from this fusion scheme and considering the YOLO-Tiny backbone architecture [23]. Compared to YOLO4-Middle, YOLO4-Tiny-Middle has a lighter backbone and only two levels in the SPP pyramid, resulting in fewer network parameters and thus faster inference generation. The complete architecture of the YOLO4-Tiny-Middle is presented in Figure 9.

The average $mAP_{50}$ obtained for the YOLO4-Tiny-Middle was 0.557 (0.639 for day and 0.491 for a night), which is less than either of the previous fusion networks. The lower mAP performance comes with a significant processing speedup compared to the previous fusion approaches, resulting in approximately 410 fps on Nvidia RTX 3080 after optimizing the model with the TensorRT library.

### 5.6. Comparison to the State-of-the-Art

Although the main aim of our research was to investigate how the fps of a deep learning detector influences its ability to detect a vulnerable road user within a sufficient distance to allow for breaking. We also draw brief comparisons between the performance of our best model, YOLO4-Middle, and other YOLO-based detectors that were published recently. While most of the works concerning multispectral pedestrian detection adopt the log average miss rate over the range of $\left[10^{-2},10^{0}\right]$ false positives per image as the performance measure, there seem to be considerable differences in the preparation of the datasets employed for the benchmarks. The KAIST dataset [9] is used most commonly, but many works adopt modified annotations, introduced in [34] and known as the “sanitized” version, because numerous annotation errors have been removed. Furthermore, some authors test their neural models on a “reasonable” setting of the KAIST dataset, using only human silhouettes taller than 50 pixels. As our research is aimed at real-world applications to autonomous vehicles, where various errors in data annotation can happen easily, and all objects endangering the vehicle should be detected, we used the KAIST dataset without any modifications. Moreover, we treated all the annotated classes as vulnerable road users that should be detected; however, these objects could also be cyclists, groups of people, or uncertain objects labeled as “person?”. Using the raw dataset certainly made the detection task harder and lowered the sore of our method, but we believe that such settings were necessary to make our breaking distance analysis fair with respect to real-world conditions.

Multispectral Channel Feature Fusion from [27] scored with its best variant the log average miss rate of 20.64% for the day and night scenarios (using “sanitized” annotations), while achieving 30 fps. A recent method based on YOLOv4 [37] reported 16 fps and the log average miss rate of 34.11% for the day and night scenarios. However, in this case it is unclear if the corrected annotations and/or “reasonable” settings were employed. In comparison, our best neural network model, YOLO4-Middle, achieved the log average miss rate of 35.81% for the day and night scenarios, with a slightly lower miss rate (34.0%) for the daytime only scenario, and a slightly worse score for the nighttime images (36.43%). Although these scores are worse than those reported in [27], they were achieved on the entire KAIST dataset, thereby including examples with very small pedestrian silhouettes, cyclists etc., and without removing the erroneous annotations. Under these conditions, our results are still on par with the log average miss rate reported on the KAIST dataset by the recent paper [37], whose authors also adopted YOLOv4. Our detector achieved more than twice faster processing though (35 fps). Compared to older two-stage detectors based on the R-CNN architecture, we achieved inference times one order of magnitude shorter, along with better detection results (e.g., [8] reports the miss rate of 36.99% using a conceptually similar fusion scheme). We consider low latency inference a key factor in achieving the ability to protect vulnerable road users from accidents in real-world automotive scenarios, and we provide results that support this statement in the next section.

## 6. Real-World Application Viability

An analysis of the practical viability of the proposed pedestrian detection method has to capture the behavior of the system in typical driving scenarios. Measuring the performance of the pedestrian detector with mean average precision does not illustrate how well the system would perform once deployed in an actual autonomous car. Therefore, we decided to simulate a typical scenario when a person appears on the road, and the car has to either brake or has to perform an avoidance maneuver to avoid a collision. In this scenario, we can estimate the performance of the pedestrian detection system depending on the inference time needed to process a single image, the speed of the vehicle, and the distance to the detected object. Unfortunately, the KAIST dataset lacks the depth information to infer the performance based on the distance to the obstacle and vehicle speed for each image. In order to do our simulation, we had to make several assumptions:

- We assumed that each detected person object represents only one person of consistent height and width;
- We assumed that each object detection was independent;
- We assumed that the detection rate was not limited by the camera’s frame rate;
- We focused on recall without the consideration for false positives.

Therefore, the following analysis is only valid if these assumptions are held and do not cover the infinite number of cases that might occur while driving.

### 6.1. Distance to the Obstacle Based on Bounding Box from the Detection

The following processing step of our simulation involved estimating the relation between the size of the bounding box measured in pixels and a metric distance to the obstacle. In our simulation, we assumed the supposed worldwide average height of an adult, approximately 1.7 m, and the average width of 0.4 m [40]. For simplicity, we assumed these dimensions for all objects marked as a person, group of people, or a person riding a bicycle. We believe it is a reasonable assumption, as instances of the latter classes rarely appear in the dataset, constituting less than 4% of all pedestrian objects.

With the knowledge that the vertical field of view of the camera was equal to θ=39° [9], we formulated an equation to compute the camera focal length of a standard pinhole camera model valid for images of size 416×416 pixels:

(1) $$f=\frac{w}{2\tan\left(\frac{\theta }{2}\right)},$$

where *w* is the width of the image in pixels. Our thermal and RGB cameras have isotropic lenses and can be modeled with a pinhole camera model to approximate the same focal length $f=f_{x}=f_{y}$ for both x and y axes. The relation computed from Equation (1) is described with the equation:

(2) $$d=f\sqrt{\frac{p_{w}p_{h}}{b}},$$

where $p_{w}$ stands for person’s width, $p_{h}$ stands for person’s height $p_{w}$, and *b* is the size of the bounding box measured in pixels. For the KAIST dataset, we found out that the pedestrian detections occur for bounding boxes *b* ranging from 50 to 5000 pixels.

**Figure 10:**
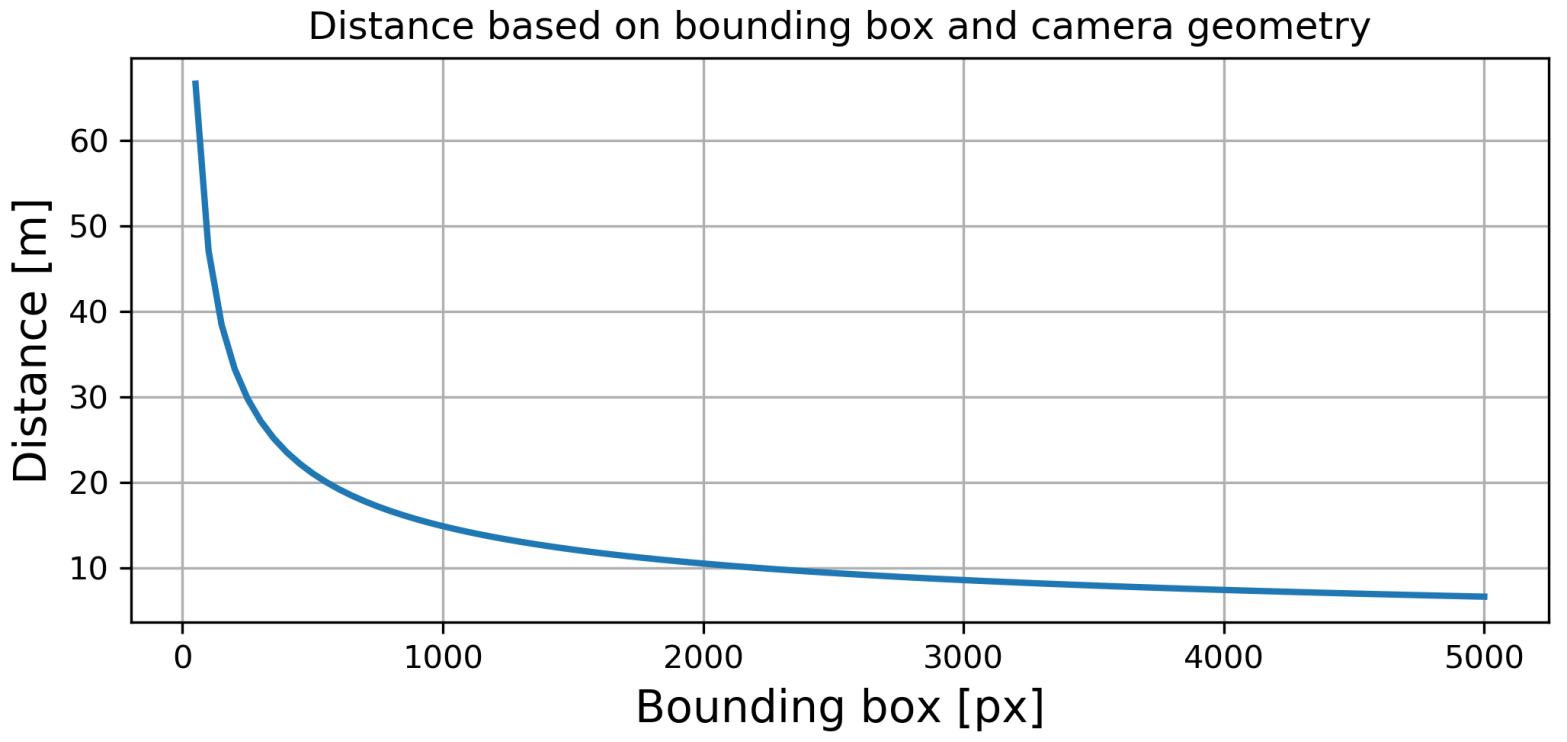
The distance to the person, based on the size of the bounding box, is registered on 416×416 pixel images.

Based on the prior assumptions, for a selected bounding box size *b*, we were able to roughly estimate the distance from the camera to the pedestrian. The relation between the bounding box size *b* and the distance to the pedestrian *d* is presented as a blue line in Figure 10. As we can see, all objects with bounding boxes exceeding approximately 500 pixels were closer than 20 m to the camera. This relation is not linear. The distance grows exponentially for smaller bounding boxes, thereby showing the importance of detecting small objects.

### 6.2. Recall as a Function of Distance

This section reports the performance of the method based on the distance. In our simulation, we assumed that the person was standing on the road, and the goal was to detect the person and brake before the collision occurred. Therefore, we were interested in ensuring that the person was consistently detected (recall), ignoring the number of false positives at this time. The recall measure was computed as:

(3) $$r=\frac{TP}{TP+FN},$$

where *TP* is the sum of all true positives defined as correct detection with an IoU measure above the defined threshold of 0.50, and *FN* is the sum of all false negatives defined as the sum of detections that should have been but were not made by the proposed algorithm.

**Figure 11:**
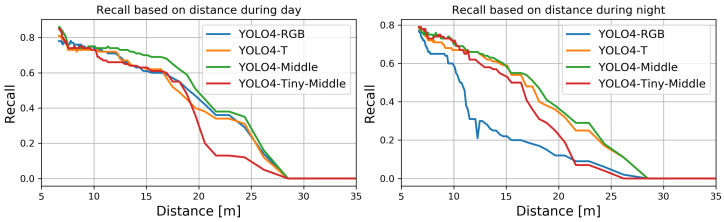
The recall for YOLO4-RGB, YOLO4-T, YOLO4-Middle, and YOLO4-Tiny-Middle as a function of distance (sizes of bounding boxes).

We computed the recall measure for each analyzed fusion method based on ground truth and estimated bounding boxes. Like the previous computations, we did them for different sizes of the ground truth bounding boxes used to determine the recall depending on the distance to the detected objects. The results for day and night conditions are presented in Figure 11.

None of the methods could enable detection at distances exceeding 28 m. For all methods, the average recall dropped almost linearly with the distance of the person from the camera. The best recalls were found for YOLO4-Middle for almost all considered distances for the day and the night. For daytime, the worst performance for more considerable distances was observed for YOLO4-Tiny-Middle, which struggle with recognition of small bounding boxes. The worst performance was the nighttime detection by YOLO4-RGB, which performed poorly for distances larger than approximately 10 m.

### 6.3. Emergency Braking Procedure

Based on the computed recalls and chosen vehicle speed, we were interested in a simulation to determine the safe driving speed with which a pedestrian detection system could initiate the emergency braking procedure and avoid a collision. More precisely, for a selected vehicle speed, we were interested in finding the distance from the sensors where our system produces correct pedestrian detection with the required 0.99 probability. The further the detection distance, the better the method performs, as the system has more time to break or perform an avoidance maneuver. We assumed that each image processing was independent during our simulation, and started off by dividing the feasible detection ranges into intervals of 50 pixels, defined by bounding box sizes, with objects sizes of 0 px ($b_{0}$), 50 px ($b_{1}$), etc., until 5000 px ($b_{100}$). For each bounding box interval, we computed the number of feasible detections:

(4) $$n_{i}=\frac{d\left(b_{i}\right)-d\left(b_{i-1}\right)}{v}f,$$

where $n_{i}$ is the sought number of detections that can be performed in the *i*-th interval that ranges for metric distances $d(b_{i-1})$ to $d(b_{i})$ based on the pixel sizes of the objects $\left(b_{i-1},b_{i}\right)$. The computation was performed for a selected vehicle speed *v* and the fps of the chosen detector *f*. Then, we were able to compute the probability of detecting a person in the selected interval of bounding box sizes (distances) with index *i*, using equation:

(5) $$p_{i}=1-{\left(1-r_{i}\right)}^{n_{i}},$$

where $p_{i}$ is the probability of object detection (recall) in the selected range and $r_{i}$ is the recall of a single detection algorithm. The accumulated probability of detection from far away to closer distances can be written as:

(6) $$q=1-\prod_{i}\left(1-p_{i}\right).$$

**Figure 12:**
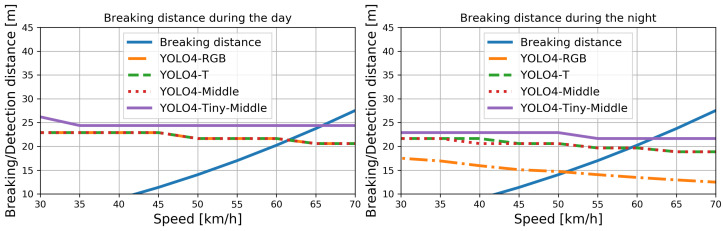
Detection distances for each method when a person can be detected with a probability exceeding 0.99. We show the typical braking distance, proving that the system can perform emergency braking when the speed does not exceed 50 km/h.

In practice, we looked for the furthest distance whereat the detection probability exceeds 0.99:

(7) $$i^{*}=\underset{j}{\mathrm{argmin}}\left(q_{j}>0.99\right)=\underset{j}{\mathrm{argmin}}\left(\left(1-\prod_{i}^{j}\left(1-p_{i}\right)\right)>0.99\right),$$

where $i^{*}$ is the ID of the detection interval defined by its bounding box size, which was used to determine the furthest detection distance $d\left(b_{i^{*}}\right)$ at which the given system obtained the required 0.99 probability of pedestrian detection. The computed detection distances for YOLO4-RGB, YOLO4-T, YOLO4-Middle, and YOLO4-Tiny-Middle for the day and night are presented in Figure 12.

As it turns out, the detection distances for all of the presented methods during the day were similar to the best performance (largest detection distance) observed for YOLO4-Tiny-Middle. During the day, YOLO4-Tiny-Middle can safely brake when driving at a speed of approximately 65 km/h, and the other solutions could work safely at about 60 km/h. The more significant differences between the methods can be observed for the nighttime attempts: YOLO4-Tiny-Middle performed best and YOLO4-RGB worst. The difference in speed for when we can avoid a collision using these two methods is approximately 11 km/h (62 vs. 51 km/h). Naturally, the person could be detected with the required probability from a greater distance (greater detection distance) with lower vehicle speeds by all considered methods.

### 6.4. Accumulated Recall Measure for Real-World Viability

**Figure 13:**
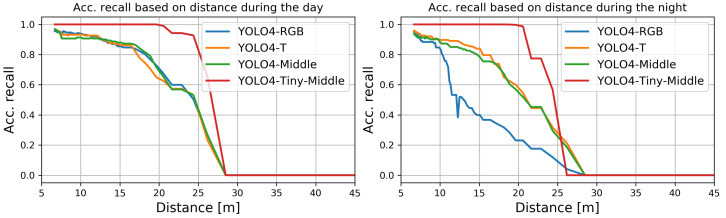
Comparison using the accumulated recall measure over 50 ms. The differences between methods are largely negligible, as better recall results were mostly obtained by more sophisticated methods that are slower.

The lack of clear visual distinction between presented methods suggests that despite the differences in recall performance, the amount of fps is the critical feature determining the performance of a solution. This proves that YOLO4-Tiny’s short inference time and high fps make it an excellent algorithm for pedestrian detection. Based on this observation, we also propose introducing a performance measure that combines the recall of a method with the feasible fps. We call this measure the accumulated recall over time:

(8) $$AccR_{t}=1-{\left(1-r\right)}^{f(t)},$$

where $AccR_{t}$ is the proposed measure metric, *r* is the recall for a single detection, and f(t) is the number of detections that can be performed in *t* milliseconds. This measure captures the recall of the method in the chosen time interval, assuming independence for all of the detections. For our further considerations, we chose $AccR_{50}$, which is measured in 50 milliseconds, and Figure 13 presents the accumulated recall measure computed for each of the analyzed solutions.

It can be seen that due to the high speed (above 35 fps) of all considered methods, their average recall performances are quite similar, with the exception of YOLO4-Tiny-Middle. Despite lower mean average precision, the high fps makes it the best method during the day. The best performance during the night was by YOLO4-Tiny-Middle; clearly worse performance was obtained for YOLO4-RGB (the right side of Figure 13).

If we consider the total accumulated recall for all detections regardless of the distances, the best performance measured using $AccR_{50}$ was obtained by YOLO4-Tiny-Middle $\left(0.999\right)$. The second best results were obtained by YOLO4-Middle $\left(0.870\right)$, followed by YOLO4-T $\left(0.866\right)$ and YOLO4-RGB $\left(0.723\right)$. Based on these measures, we see that the method’s performance should be analyzed together with its speed, and YOLO4-Middle outperformed YOLO4-RGB and YOLO4-T according to this measure. Moreover, efficient data fusion can also be performed with a lightweight network, thereby achieving excellent performance day and night.

## 7. Discussion and Conclusions

In this article, we considered the problem of low latency detection of the pedestrians from pairs of co-registered RGB and thermal images. Unlike many other approaches, we focused on the real-world viability of the method in scenarios related to autonomous driving.

From the set of different approaches based on a single modality (YOLO4-RGB, YOLO-T) or both modalities (YOLO4-HST, YOLO4-GST, YOLO4-RGB-T, YOLO4-Middle, YOLO4-Late), we determined that the fusion performed in the middle stage of the YOLO detector processing pipeline (YOLO4-Middle) provides the best performance during the day and at night, which corroborates earlier results from the literature [8]. The YOLO4-Middle detector performs better than YOLO4-RGB during the daytime and better than YOLO4-T during the night, regardless of the object size.

Measuring the mAP and/or log average miss rate scores on a public dataset is usually the final step in other works concerning this problem. We went further, by proving that YOLO4-Middle can process over 35 images per second on the desktop RTX 3080 GPU. However, as automotive applications call for lower-end or embedded GPUs, we aimed to further improve the processing speed with YOLO4-Tiny-Middle, a lightweight implementation of our best YOLOv4-based fusion architecture. Although with this architecture we achieved slightly lower $mAP_{50}$ and recall rates, the inference time of YOLO4-Tiny-Middle is an order of magnitude shorter than that of the full-scale version.

Measuring the mAP and fps is not enough to verify a solution for real autonomous cars. Therefore, we measured the performance in a simulated scenario in which a pedestrian emerged in front of the car, and the system had to brake to avoid a collision. From this analysis on the KAIST dataset, it can be seen that high-fps detectors outperformed their more classic counterparts, due to their ability to perform more frame processing passes within the same time window. We also proposed an “accumulated recall” measure that jointly captures recall and fps based on this information. Accumulated recall is suited to capturing the real-world feasibility of pedestrian detectors. Hence, we argue that this new measure can be used to assess the readiness of detectors when product deployment is considered.

Our experiments proved that thermal and visible light images can be fused in the modern YOLOv4 architecture without additional overheads in processing, to achieve very high fps processing, as long as both images are synchronized and aligned. These assumptions are somewhat limiting, as no out-of-the-box co-aligned RGB and thermal cameras are available for automotive applications. Therefore, we see two exciting branches for further research. The first will focus on utilizing data streams from both modalities when the images are not co-aligned, making a practical solution when two separate cameras are combined in a single sensory setup. The other direction of future research will investigate the detection of small objects in images and the addition of preprocessed LiDAR data [41] for better detection of pedestrians from long distances.

## Figures and Tables

**Table 1:** Division of the KAIST dataset into the training, validation, and test sets according to the time of day based on the number of images and number of annotated ground truth objects.

	Training		Validation		Test		Total	
	Img.	Obj.	Img.	Obj.	Img.	Obj.	Img.	Obj.
Day	41 k	54 k	13 k	12 k	8 k	4 k	62 k	70 k
Night	20.5 k	36 k	9 k	5 k	3.5 k	3.5 k	33 k	44.5 k
Total	61.5 k	90 k	22 k	17 k	11.5 k	7.5 k	95 k	114.5 k

**Table 2:** The obtained mean average precision $mAP_{50}$ metric for different types of pedestrian detection systems depending on the time of day. The best result for the selected time of the day is marked in blue, and the worst is red.

	Time of Day		
	Day	Night	Day + Night
YOLO4-RGB	0.684	0.298	0.465
YOLO4-T	0.641	0.617	0.625
YOLO4-HST	0.672	0.62	0.639
YOLO4-GST	0.673	0.603	0.627
YOLO4-RGB-T	0.648	0.609	0.618
YOLO4-Middle	0.751	0.626	0.686
YOLO4-Late	0.666	0.636	0.645

**Table 3:** Model inference expressed in fps for all of the YOLOv4 detectors.

	Original [fps]	Optimized [fps]	Original Single	Optimized Single
			Inference Time [ms]	Inference Time [ms]
YOLO4-RGB	28.1	41.0	35.6	24.4
YOLO4-T	28.1	41.0	35.6	24.4
YOLO4-HST	27.9	39.9	35.8	25.1
YOLO4-GST	27.8	39.8	36.0	25.1
YOLO4-RGB-T	27.6	39.6	36.2	25.3
YOLO4-Middle	21.7	35.2	46.1	28.4
YOLO4-Late	27.1	38.2	36.9	26.2

## Data Availability

The code used in this manuscript is available on GitHub (https://github.com/KRoszyk/YOLOv4-Middle_detector accessed on 26 January 2022) and can be cited using Zenodo (https://zenodo.org/record/5899548#.Ye8mAXVKhhE accessed on 26 January 2022). The used KAIST dataset is available on GitHub (https://github.com/SoonminHwang/rgbt-ped-detection accessed on 15 January 2021).
